# Complement Activation and Organ Damage After Trauma—Differential Immune Response Based on Surgical Treatment Strategy

**DOI:** 10.3389/fimmu.2020.00064

**Published:** 2020-01-31

**Authors:** Ina Lackner, Birte Weber, Meike Baur, Giorgio Fois, Florian Gebhard, Roman Pfeifer, Paolo Cinelli, Sascha Halvachizadeh, Miriam Lipiski, Nikola Cesarovic, Hubert Schrezenmeier, Markus Huber-Lang, Hans-Christoph Pape, Miriam Kalbitz

**Affiliations:** ^1^Department of Traumatology, Hand-, Plastic- and Reconstructive Surgery, University of Ulm, Ulm, Germany; ^2^Institute of General Physiology, University of Ulm, Ulm, Germany; ^3^Department of Trauma, University Hospital of Zurich, Zurich, Switzerland; ^4^Department of Surgical Research, University Hospital of Zurich, Zurich, Switzerland; ^5^Institute of Transfusion Medicine, University of Ulm and Institute of Clinical Transfusion Medicine and Immunogenetics Ulm, Ulm, Germany; ^6^German Red Cross Blood Transfusion Service Baden-Württemberg – Hessen and University Hospital Ulm, Ulm, Germany; ^7^Institute for Clinical- and Experimental Trauma-Immunology, University of Ulm, Ulm, Germany

**Keywords:** multiple trauma, cardiac dysfunction, femoral nailing, conventional reaming, inflammation

## Abstract

**Background:** The complement system is part of the innate immunity, is activated immediately after trauma and is associated with adult respiratory distress syndrome, acute lung injury, multiple organ failure, and with death of multiply injured patients. The aim of the study was to investigate the complement activation in multiply injured pigs as well as its effects on the heart *in vivo* and *in vitro*. Moreover, the impact of reamed vs. non-reamed intramedullary nailing was examined with regard to the complement activation after multiple trauma in pigs.

**Materials and Methods:** Male pigs received multiple trauma, followed by femoral nailing with/without prior conventional reaming. Systemic complement hemolytic activity (CH-50 and AH-50) as well as the local cardiac expression of C3a receptor, C5a receptors1/2, and the deposition of the fragments C3b/iC3b/C3c was determined *in vivo* after trauma. Human cardiomyocytes were exposed to C3a or C5a and analyzed regarding calcium signaling and mitochondrial respiration.

**Results:** Systemic complement activation increased within 6 h after trauma and was mediated via the classical and the alternative pathway. Furthermore, complement activation correlated with invasiveness of fracture treatment. The expression of receptors for complement activation were altered locally *in vivo* in left ventricles. C3a and C5a acted detrimentally on human cardiomyocytes by affecting their functionality and their mitochondrial respiration *in vitro*.

**Conclusion:** After multiple trauma, an early activation of the complement system is triggered, affecting the heart *in vivo* as well as *in vitro*, leading to complement-induced cardiac dysfunction. The intensity of complement activation after multiple trauma might correlate with the invasiveness of fracture treatment. Reaming of the femoral canal might contribute to an enhanced “second hit” response after trauma. Consequently, the choice of fracture treatment might imply the clinical outcome of the critically injured patients and might be therefore crucial for their survival.

## Introduction

The complement system is part of the innate immunity and is activated immediately after trauma in response to pathogen- and damage-associated molecular patterns (PAMPs and DAMPs) (1). During complement activation, structural rearrangements, proteolytic cleavage, and the assembly of proteolytic and lytic complexes occur in a complex signal cascade, leading finally to the destruction and elimination of pathogens (2). In multiply injured patients, the complement system is rapidly activated after polytrauma, leading to the so-called trauma-induced “complementopathy” shortly after injury. This early complementhopathy is accompanied with a massive systemic release of the anaphylatoxins C3a and C5a in serum of multiply injured patients, triggering the innate immune response after severe trauma (3). C3a as well as C5a plasma concentrations correlate with the severity of trauma and are predictors for the development of the acute respiratory distress syndrome (ARDS) and of multiple organ failure (MOF), which both often occur after multiple trauma in humans (3–9). Moreover, the development of acute lung injury (ALI) is described in critically injured patients as well as in animals after experimental trauma and is also linked to the activation of the complement system (10–13). In critically injured patients, the early activation of the complement system is mostly mediated via the alternative pathway, correlating with injury severity and with worse clinical outcomes (14). Moreover, complement activation via the classical pathway was also described after trauma and was further associated with an increased mortality rate of the patients (15).

In patients with femur shaft fracture internal fixation by intramedullary nailing is the gold standard (16). Reaming of the femoral canal allows the insertion of a nail with bigger diameter and is further associated with the release of endogenous osteogenic factors in the reaming debris (17). Additionally, the mechanical stability is improved by bigger diameter of the inserted nail (18). Reamed intramedullary nailing is associated with shorter time to union and with lower rates of delayed-union, non-union, and reoperation (19). Nevertheless, femoral reaming has been reported to prime the immuno-inflammatory response, leading to the “second hit” phenomenon in multiply injured patients (17, 20–22). Furthermore, femoral reaming has been linked to post-traumatic complications such as ARDS and multiple organ dysfunction syndrome (MODS) (20–22). The concept of damage control orthopedics (DCO) in patients with multiple trauma and especially in presence of blunt chest trauma is based on these findings (23). With regard to cardiopulmonary consequences during femoral reaming, increased intramedullary pressure has been described. Increased intramedullary pressure was associated with intravasation of bone marrow contents, leading to bone marrow embolization in the lungs, which was further associated with altered cardiopulmonary function (24).

Besides diverse complement-induced organ damage, the activation of the complement system is strongly involved in cardiac pathology. The anaphylatoxin C5a was shown to be strongly cardio-depressive by acting via the C5aR1/2 on the membrane of cardiomyocytes (CMs), inducing defective CM contractility, reduced cardiac output, leading finally to cardiac dysfunction (25–27). Absence of either C5aR1 or C5aR2 attenuates cardiac dysfunction and improves survival of mice during sepsis (28). Moreover, C5a triggers a massive amount of cytosolic reactive oxygen species (ROS) and intracellular calcium [Ca^2+^i] in isolated rat CMs (28). Enhanced ROS was associated with cardiac remodeling, reduced left ventricular (LV)-function as well as with contractile dysfunction (29, 30). The increased [Ca^2+^i] affected the homeostasis and the electrophysiological functions of the CMs (28). Moreover, C5a induces defects in CMs contractility and relaxation by altering and disturbing their action potentials, nominating C5a as very powerful cardio-suppressive factor (25, 28). C3a was also shown to be cardio-depressive, leading to cardiac dysfunction, arrhythmia and contractile failure (31). Nevertheless, C3a and C5a seem also crucial during systemic inflammation by enhancing pro-inflammatory and inflammatory danger signaling pathways as well as exhibiting direct antimicrobial effects (32, 33).

In this study, we aim to investigate the early activation of the complement system in multiply injured pigs within the first 6 h after trauma with a focus on the heart. Thereby, systemic complement activation should be examined more detailed with respect to different surgical treatment strategies (reamed vs. non-reamed intramedullary nailing). Moreover, the expression of the receptors for complement activation (C3aR, C5aR1/2) as well as the deposition of C3 cleavage products C3b/iC3b/C3c should be analyzed locally in left ventricular cardiac tissue *in vivo*. Further, we aim to thoroughly investigate the effects of C3a and C5a on human CMs *in vitro*. The goal of the study was to get a closer understanding of the underlying molecular mechanisms leading to complement-induced cardiac dysfunction in the context of multiple trauma.

## Materials and Methods

### Animals

This study presents results obtained from a project using porcine multiple trauma model, conducted by the TREAT research group.

The animal housing and experimental protocols were approved by the Cantonal Veterinary Department, Zurich, Switzerland, under license no. ZH 138/2017, and were in accordance with Swiss Animal Protection Law and Ordinance. Housing and experimental procedures also conformed to the European Directive 2010/63/EU of the European Parliament and of the Council on the Protection of vertebrate animals used for scientific purposes (Council of Europe no. 123, Strasbourg 1985) and to the Guide for the Care and Use of Laboratory Animals (Institute of Laboratory Animal Resources, National Research Council, National Academy of Sciences, 2011).

25 male pigs weighting 50 ± 5 kg (*Sus scrofa domestica*) were included in the study (mean height: 123.6 cm). General instrumentation, anesthesia, and trauma induction were described previously by Horst et al. (34). Animals were held in controlled environment with 21 ± 3°C room temperature (50% humidity), with a light/dark cycle of 12 h. Water was available for animals *ad libitum*.

### Multiple Trauma in Pigs

In the present study, 15 pigs were investigated. Pigs underwent either multiple trauma (*n* = 10) or sham-procedure (*n* = 5). Multiple trauma includes a combination of a penetrating thorax trauma, laparotomy, liver laceration, femur fracture, and hemorrhagic shock (ISS ≥ 27). Control animals underwent sham-procedure (*n* = 5). Femur fracture was induced by a bolt gun (Blitz-Kernen, turbocut JOBB GmbH, Germany), positioned on the mid third of the left femur. For introduction of blunt chest trauma, a pair of panels (steel 0.8 cm, lead 1.0 cm thickness) was placed on the right dorsal lower chest. A shock wave was induced by a bolt shot (Blitz-Kerner, turbocut JOBB GmbH, Germany), which was applied onto the panel using cattle-killing cartridges as previously described (35, 36). Midline-laparotomy was performed by exploring the right upper liver lobe. Penetrating hepatic injury was induced by cross-like incision halfway through the liver tissue. After a short period of uncontrolled bleeding (30 s), liver package was performed. Directly after hepatic packing, pressure-controlled and volume-limited hemorrhagic shock was induced by withdrawing of blood until a mean arterial pressure (MAP) of 30 ± 5 mm Hg was reached. Maximal withdrawal amounts to 45% of total blood volume. The reached MAP was maintained for 60 min. At the end of the shock period, animals were resuscitated according to established trauma guidelines (ATLS®, AWMF-S3 guideline on Treatment of Patients with Severe and Multiple Injuries®) by adjusting FiO_2_ and an initial substitution of the withdrawn blood volume with Ringerfundin. Fluid maintenance was performed by infusion of additional fluids (Ringerfundin, 2 ml/kg body weight/h). Further, pigs were rewarmed until normothermia (38.7–39.8°C) was reached. Sham procedure (*n* = 5) included instrumentation and anesthesia but without trauma or hemorrhage. The multiple trauma group (*n* = 10) was randomized in two therapy arms: pigs received either femoral nailing without reaming (*n* = 5) or standard reaming (*n* = 5). In both groups, a shortened conventional tibia nail was introduced.

### Follow-Up and Euthanasia

Hemodynamic parameters were continuously monitored for 6 h. Pigs were euthanized under deep, general anesthesia with intravenous Na-Pentobarbital.

### Sample Collection

Serum and plasma samples were collected at baseline, 4 and 6 h after multiple trauma and kept on ice. After centrifugation (1,500 g for 12 min at 4°C), serum and EDTA-plasma were removed and stored at −80°C until analysis. Heart tissue samples were obtained 6 h after resuscitation. Tissue of the superficial and the luminal left ventricle was fixed with 4% formalin, followed by embedding in paraffin. Furthermore, tissue was quick-frozen in liquid nitrogen, followed by storage at −80°C until analysis.

### Transesophageal Echocardiography in Pigs

Imaging was performed according to the recommendations using a standard cardiac ultrasound machine (Cx50 xMATRIX, Phillips Healthcare, Germany with the X7-2t probe and the S5-1 ultrasound probe for additional transthoracic measurements). Serial imaging was performed before, 4 and 6 h after trauma by an experienced investigator for echocardiography in pigs. The ejection fraction (EF) was calculated as *EF* (%) = (*EDV*–*ESV) 1/EDV* × 100 (EDV = end-diastolic volume; ESV = end-systolic volume). Further, blood pressure curves were measured continuously over 6 h. Thereby, following parameters were determined: heart rate (HR) in beats per minute (bpm), systolic, diastolic blood pressure, and mean arterial pressure (MAP) in mmHg at trauma as well as 1, 2, 3, 4, and 6 h after trauma.

### Complement Hemolytic Activity

#### Classical Pathway (CH-50)

Sensitized sheep erythrocytes (Complement Technology Inc., Tyler, TX, USA) were washed once with tris buffered saline (TBS), centrifuged (3 min, 4°C, 500 g) and erythrocytes were re-suspended in GVB^++^ buffer (with Ca^2+^ and Mg^2+^, pH 7.3) (Complement Technology Inc., Tyler, TX, USA). GVB^++^ buffer contains 0.1% gelatin, 5 mM Veronal, 145 mM NaCl, 0.025% NaN_3_, 0.15 mM CaCl_2_, and 0.5 mM MgCl_2_, which allows the process of complement activation via the classical pathway. In order to determine 50 and 100% lysis, the erythrocytes were diluted with ddH_2_O. The optical density was measured at 415 nm and OD values for 50 and 100% lysis were determined.

Serial dilution of porcine serum from 1:20 through 1:640 was prepared in GVB^++^ buffer (with Ca^2+^ and Mg^2+^, pH 7.3). Then, sensitized sheep erythrocytes were added to the diluted serum samples and incubated for 30 min at 37°C. Afterwards, ice cold GVBE buffer (with EDTA, pH 7.3) (Complement Technology Inc., Tyler, TX, USA) was added to the samples and samples were centrifuged (3 min, 4°C, 500 g). GVBE buffer contains 0.1% gelatin, 5 mM Veronal, 145 mM NaCl, 0.025% NaN_3_, and 10 mM EDTA, which inhibits the complement activation cascade. Afterwards, the supernatant was transferred and optical density from supernatant was measured at 415 nm.

#### Alternative Pathway (AH-50)

Sensitized rabbit erythrocytes (Complement Technology Inc., Tyler, TX, USA) were washed once with tris buffered saline (TBS), centrifuged (3 min, 4°C, 500 g) and erythrocytes were re-suspended in GVB^0^ buffer (without Ca^2+^ and Mg^2+^, pH 7.3) (Complement Technology Inc., Tyler, TX, USA). GVB^0^ buffer contains 0.1% gelatin, 5 mM Veronal, 145 mM NaCl, and 0.025% NaN_3_. GVB^0^ is a basic buffer which can be used to make other traditional buffers for complement assays. In order to determine 50 and 100% lysis, the erythrocytes were diluted with ddH_2_O. The optical density was measured at 415 nm and OD values for 50 and 100% lysis were determined.

Serial dilution of porcine serum from 1:2 through 1:32 was prepared in GVB^0^ buffer with 5 mM MgEGTA. The addition of MgEGTA allows the process of complement activation via the alternative pathway. Then, sensitized rabbit erythrocytes were added to the diluted serum samples and incubated for 30 min at 37°C. Afterwards, ice cold GVBE buffer (with EDTA, pH 7.3) (Complement Technology Inc., Tyler, TX, USA) was added to the samples and samples were centrifuged (3 min, 4°C, 500 g). GVBE buffer contains 0.1% gelatin, 5 mM Veronal, 145 mM NaCl, 0.025% NaN_3_, and 10 mM EDTA, which inhibits the complement activation cascade. Afterwards, supernatant was transferred and optical density from supernatant was measured at 415 nm.

### Immunohistochemical Staining (IHC)

Paraffin sections of left ventricles were dewaxed and rehydrated in a descending series of ethanol. Antigen unmasking was performed by boiling sections at 100°C in 10 mM citrate buffer (pH 6). Unspecific binding sites were blocked with 10% goat serum. Specific antigen binding was performed by incubating sections with the respective primary antibody for C3a receptor (C3aR) (Abcam, Cambridge, UK), C5a receptor 1 (C5aR1) (Acris, Rockville, MD, USA), and cleaved C3 fragments C3b/iC3b/C3c (HycultBiotech, Wayne, PA, USA) for 1 h at RT. Specific antibody binding was detected by using DakoREAL™ Alkaline Phosphatase/RED Detection System (Agilent Technologies, Santa Clara, CA, USA). Cell nuclei were counterstained with Hematoxylin. Sections were investigated by bright field microscopy using Axio Imager M.2 microscope and the Zeiss ZEN 2.3 software (Zeiss, Jena, Germany). Results are presented as mean pixel density. The levels of C3aR, C3b/iC3b/C3c, and C5aR1 in porcine samples were determined by IHC due to the restricted availability of tools.

### RNA Isolation From Heart Tissue

RNA from left ventricles was isolated using TRIzol RNA isolation reagent (ThermoFisher, Waltham, MA, USA).

### RNA Isolation From Cell Lysates

RNA isolation from cell lysates was performed by using ISOLATE II RNA Mini Kit (Meridian Bioscience, Cincinnati, OH, USA). Remaining DNA was digested by DNaseI (Meridian Bioscience, Cincinnati, OH, USA).

### Reverse Transcribed Quantitative Polymerase Chain Reaction RT-qPCR

The respective RNA samples were reverse transcribed in cDNA using SuperScript™ IV VILO™ MasterMix (Invitrogen, Carlsbad, CA, USA). For quantitative PCR the PowerUp™ SYBR™ Green Master Mix (Applied Biosystems, Waltham, MA, USA) was used. All procedures were performed according to the manufacturer's instructions. For qPCR the QuantStudio3 system (Applied Biosystems, Waltham, MA, USA) was utilized. Quantitative mRNA expression of *porcine C5a receptor-2 (C5aR2)* (forward: 5′-AAGAGATGCTCTCCTGGACCT-3′, reverse: 5′-AAACTGTGTCAGTCCGGCTC-3′) and human *heart fatty acid binding protein (HFABP/FABP3)* (forward: 5′-GCATCACTATGGTGGACGCT-3′, reverse: 5′-AACCCACACCGAGTGACTTC-3′)*, connexin43* (forward: 5′-GGCTTTTAGCGTGAGGAAAGT-3′, reverse: 5′-AAGGCAAGTTCAGGCACTCA-3′)*, sarcoplasmic/endoplasmic reticulum ATPase (SERCA2a)* (forward: 5′-CTCCTTGCCCGTGATTCTCA-3′, reverse: 5′-CCAGTATTGCAGGTTCCAGGT-3′)*, sodium-calcium exchanger (NCX)* (forward: 5′-GCCTGGTGGAGATGAGTGAG-3′, reverse: 5′-ACAGGTTGGCCAAACAGGTA-3′) was examined and calculated by the cycle threshold method ΔΔCt. Respective genes were normalized using housekeeping gene *glutaraldehyde-phosphate dehydrogenase (GAPDH)* for pig (forward: 5′-GAGTGAACGGATTTGGCC-3′, reverse: 5′-AAGGGGTCATTGATGGCGAC-3′) and for human *GAPDH* (forward: 5′-TCTCTGCTCCTCCTGTTCGAC-3′, reverse: 5′-CCAATACGACCAAATCCGTTGA-3′). Results are presented as mean fold change. The mRNA levels of C5aR2 in porcine samples were determined by qPCR due to the restricted availability of tools.

### *In vitro* Experiments

Human cardiomyocytes (iPS) (Cellular Dynamics, Madison, WI, USA) were cultured for 10 days in maintenance medium (Cellular Dynamics, Madison, WI, USA) at 37°C and in an atmosphere of 7% CO_2_. For experiments, human CMs were incubated with either 10 ng/ml C5a or with 500 ng/ml C3a (both from Merck, Darmstadt, Germany) for 6 h at 37°C in an atmosphere of 7% CO_2_.

### Viability Assay and Caspase-3/7 Assay

Cell viability of human cardiomyocytes was determined using CellTiter-Glo® Luminescent Cell Viability Assay (Promega, Madison, WI, USA) and Caspase-3/7 activity was examined by using Caspase-Glo® 3/7 Assay (Promega, Madison, WI, USA). All procedures were performed according to manufacturer's instructions.

### Calcium Measurements

For calcium measurements human cardiomyocytes (iPS) were seeded on 8-well chambers (IBIDI, Munich, Germany). Before the measurements, cells were incubated with either 10 ng/ml C5a or 500 ng/ml C3a 60 min before the start of the experiments, as well as for the duration of the experiment. For measurement of changes in intracellular Ca^2+^ concentration, cells were loaded with 5 μM Fura-2 (ThermoScientific, Waltham, MA, USA) for 30 min (in presence of pharmacological compounds if needed). After incubation, cells were washed twice with bath solution (in mM: 140 NaCl; 5.4 KCl; MgCl_2_; 1.8 CaCl_2_; 5.5 Glucose; 5 Hepes; pH = 7.4). Fluorescence imaging was performed on a Cell Observer inverse microscope with a Zeiss Fluor 40x NA 1.3 oil objective (Zeiss, Jena, Germany). Cells were illuminated for 90 ms at a rate of 2 Hz at each excitation wavelength (340 and 380 nm). Images were acquired using MetaFluor (Molecular Devices, Ismaning, Germany). Fura-2 ratios were calculated with ImageJ and the data obtained were analyzed with the Matlab script PeakCaller (37).

### Mitochondrial Respiration

Mitochondrial respiration was analyzed by using the Seahorse XFe96 Analyzer (Agilent Technologies, Santa Clara, CA, USA). For analysis of mitochondrial respiration, the Seahorse XF Cell Mito Stress Test Kit (Agilent Technologies, Santa Clara, CA, USA) was used. After the experiment, cells were fixed with 4% formalin at 4°C for overnight. Then, cells were stained with 0.2% Janus-Green solution, washed and resolved with 0.5 M hydrochloric acid. OD was measured at 630 nm. The oxygen consumption rate (OCR) values were normalized to the OD 630 nm values, respectively. Results were evaluated using Seahorse Wave 2.4 software (Agilent Technologies, Santa Clara, CA, USA).

### Immunofluorescent Staining of Human Cardiomyocytes

Human cardiomyocytes were incubated for 3 h with 10 ng/ml C5a. Afterwards, cells were washed and fixed with 4% formalin for 15 min at RT. Cells were permeabilized with 0.03% Triton-X for 10 min at RT. Unspecific binding sites were blocked with 10% goat serum. Specific antigen binding was performed by incubating cells with specific primary antibody for C5aR1 (Proteintech, Rosemont, IL, USA) for overnight at 4°C. As second antibody the AlexaFluor-488 IgG (Jackson Immunoresearch, Cambridgeshire, UK) was used. Staining of cytoskeletal actin filaments was performed by using rhodamine-labeled phalloidin (Invitrogen, Carlsbad, CA, USA) and cell nuclei were stained with Hoechst. Cells were analyzed by fluorescence microscopy using Axio Imager M.2 microscope and the Zeiss ZEN 2.3 software (Zeiss, Jena, Germany).

### Statistical Analysis

All values were expressed as means ± SEM. Data were analyzed by one-way ANOVA followed by Dunnett's or Tukey's multiple comparison test. Data of the *in vitro* experiments were also analyzed by unpaired students *t*-test. *p* ≤ 0.05 was considered statistically significant. GraphPad Prism 7.0 software was used for statistical analysis (GraphPad Software, Incorporated, San Diego, CA, USA).

## Results

### Hemodynamic and Functional Cardiac Parameters of Multiply Injured Pigs

**Figure 1:**
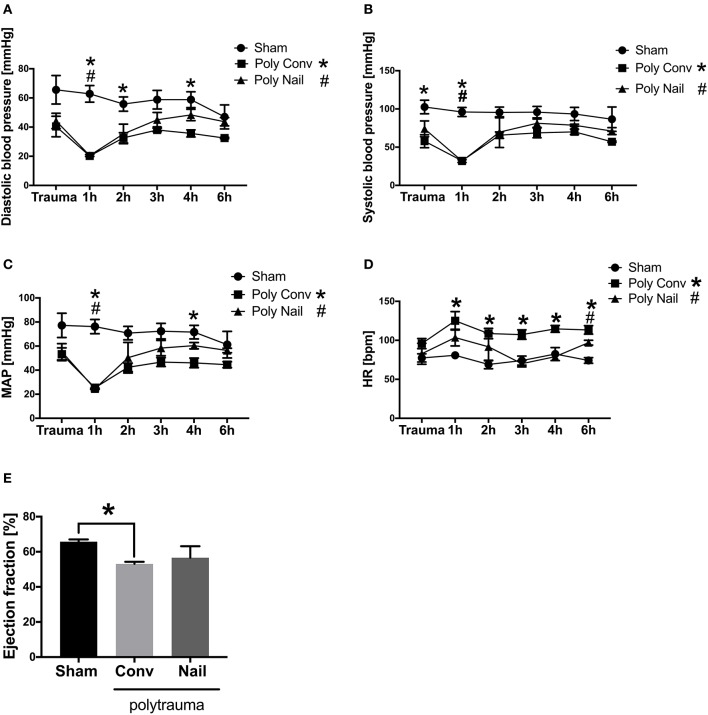
Hemodynamic and functional cardiac parameters of multiply injured pigs. Pigs received multiple injury, followed by femoral nailing of the fracture (poly nail, triangle) or conventional reaming of the fracture (poly conv, square). Control animals underwent sham-procedure (sham, circle). Blood pressure was measured continuously over 6 h and systolic blood pressure (mmHg) **(A)**, diastolic blood pressure (mmHg) **(B)**, mean arterial pressure (MAP) (mmHg) **(C)**, heart rate (HR) (bpm) **(D)** were measured directly at trauma as well as 1, 2, 3, 4, and 6 h after trauma. Cardiac ejection fraction (%) **(E)** was determined by transesophageal echocardiography 6 h after trauma. Results are presented as mean ± SEM. *n* = 5. *p ≤ 0.05.

The blood pressure of multiply injured pigs was measured at trauma as well as 1, 2, 3, 4, and 6 h after trauma. The systolic blood pressure significantly decreased at trauma as well as 1 h after trauma in the group, which received conventional reaming of the fracture compared to the sham group. In group with femoral nailing the systolic blood pressure significantly decreased 1 h after trauma compared to the sham group (Figure 1A). The diastolic blood pressure significantly decreased 1, 2, and 4 h after trauma in the group with conventional treatment of the fracture compared to the sham group. In group with femoral nailing of the fracture, the diastolic blood pressure significantly decreased 1 h after trauma compared to the sham group (Figure 1B). The mean arterial pressure (MAP) significantly decreased 1 and 4 h after trauma in group with conventional reaming compared to the sham group. In the group with femoral nailing, the MAP significantly decreased 1 h after trauma compared to the sham group (Figure 1C). The heart rate significantly increased 1, 2, 3, 4, and 6 h after trauma in the group with conventional reaming compared to the control group. In the group with femoral nailing, the heart rate significantly increased 6 h after trauma compared to the sham group (Figure 1D). The cardiac ejection fraction significantly decreased 6 h after trauma in the group, which received conventional reaming of the fracture compared to the sham group (Figure 1E).

### Complement Hemolytic Activity in Multiply Injured Pigs

#### CH-50-Classical Pathway

**Figure 2:**
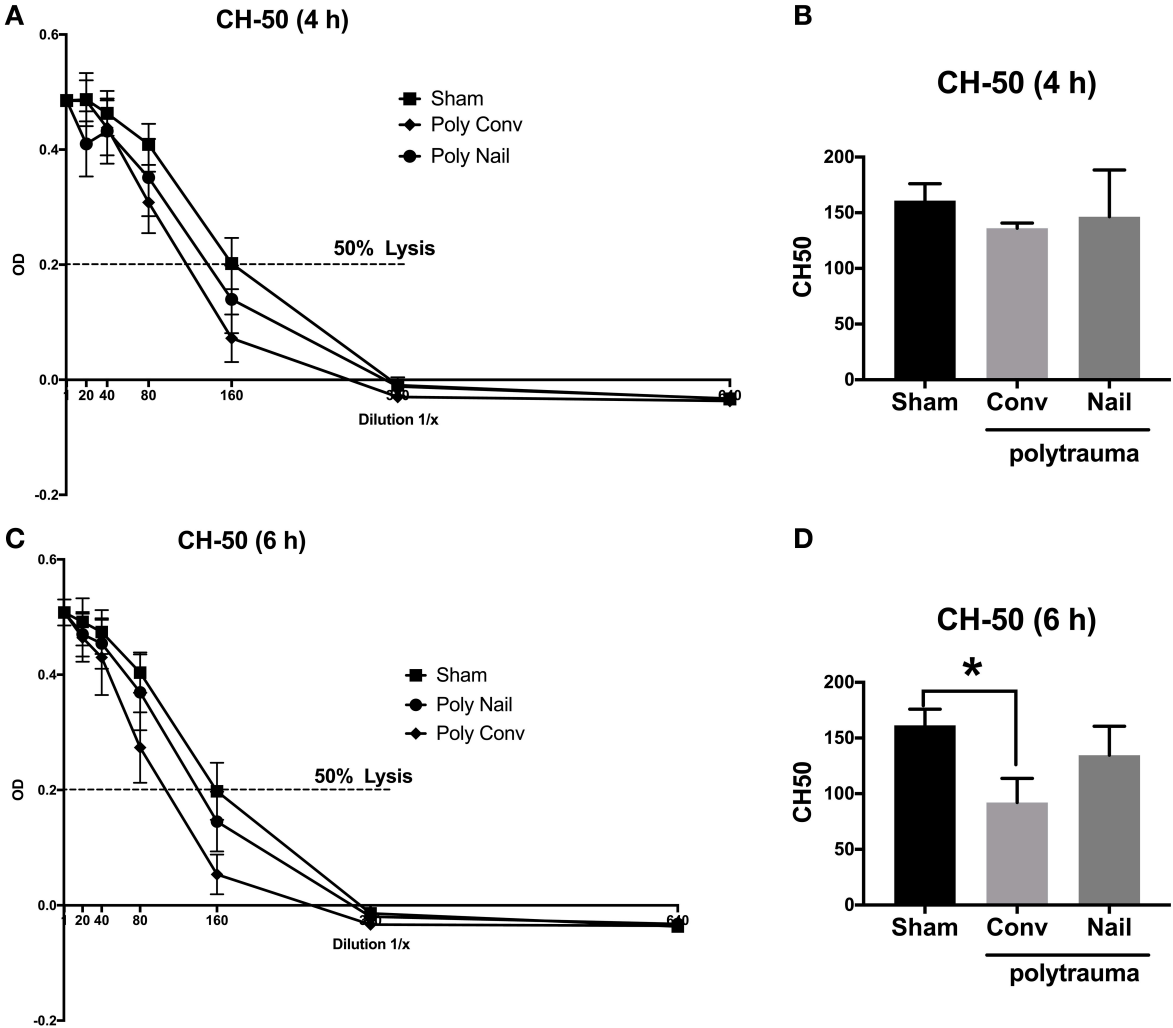
Complement hemolytic activity in serum of multiply injured pigs via the classical pathway (CH-50). Pigs received multiple injury, followed by femoral nailing of the fracture (poly nail, circle) or conventional reaming of the fracture (poly conv, rhombus). Control animals underwent sham-procedure (sham, square). CH-50 was measured at 4 h **(A,B)** and 6 h **(C,D)** after trauma. For CH-50 lysis curves, a serial dilution of the serum from 4 h after trauma **(A)** and 6 h after **(C)** was performed (x-axis, dilution in 1/x) and the respective OD values of the serum were determined at 415 nm (y-axis, OD). Dotted line represents OD value of 50% lysis of sensitized sheep erythrocytes. CH-50 values (1/x) of 4 h after trauma **(B)** and 6 h after trauma **(D)**. Results are presented as mean ± SEM. *n* = 5. *p ≤ 0.05.

In order to determine early complement activation after multiple trauma, we analyzed the complement hemolytic activity in serum of multiply injured pigs compared to the sham treated animals. Considering the classical pathway of complement activation, the CH-50 lysis curves of the two trauma groups were shifted to the left after 4 and 6 h compared to the control group, needing a higher dilution for lysis of the sensitized erythrocytes due to already consumed complement factors (Figures 2A,C). This was also demonstrated in the respective calculated CH-50 values of the CH-50 lysis curves. The CH-50 decreased within 6 h in the polytrauma group, which received internal fixation by femoral nailing (Figures 2B,D). In the polytrauma group with conventional reaming of the fracture, the CH-50 also decreased within 6 h after trauma and dropped significantly 6 h after trauma compared to the sham treated group (Figures 2B,D). Lower CH-50 values indicate for increased complement activation.

#### AH-50-Alternative Pathway

**Figure 3:**
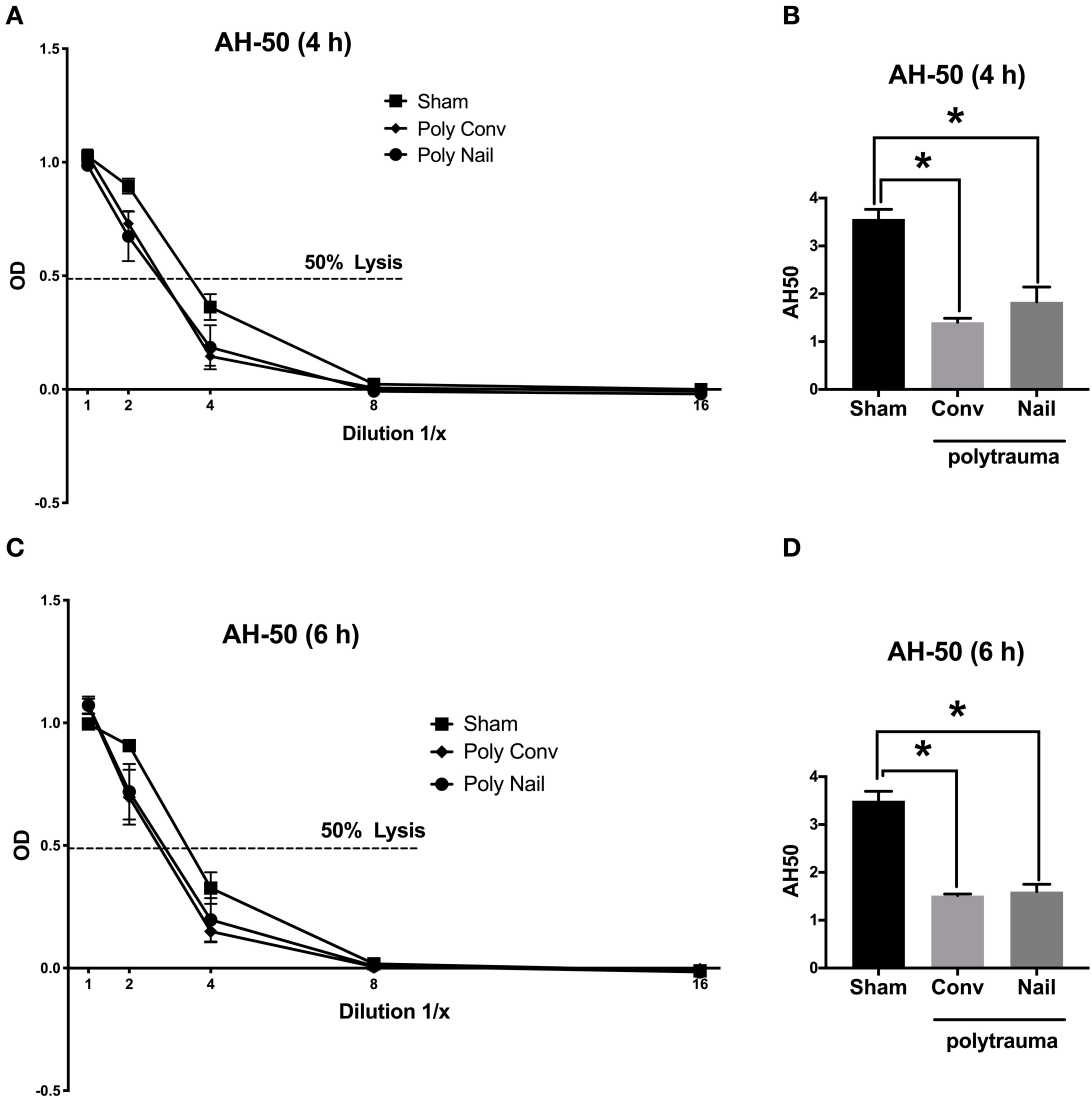
Complement hemolytic activity in serum of multiple injured pigs via the alternative pathway (AH-50). Pigs received multiple injury, followed by femoral nailing of the fracture (poly nail, circle) or conventional reaming of the fracture (poly conv, rhombus). Control animals underwent sham-procedure (sham, square). AH-50 was measured at 4 h **(A,B)** and 6 h **(C,D)** after trauma. For AH-50 lysis curves, a serial dilution of the serum from 4 h after trauma **(A)** and 6 h after **(C)** was performed (x-axis, dilution in 1/x) and the respective OD values of the serum were determined at 415 nm (y-axis, OD). Dotted line represents OD value of 50% lysis of sensitized sheep erythrocytes. AH-50 values (1/x) of 4 h after trauma **(B)** and 6 h after trauma **(D)**. Results are presented as mean ± SEM. *n* = 5. *p ≤ 0.05.

Regarding the alternative pathway, the lysis curves of the two trauma groups were shifted to the left after 4 h and 6 h compared to the control group, indicating for already consumed complement factors after trauma (Figures 3A,C). This was also reflected in the respective calculated AH-50 values of the AH-50 curves. The AH-50 decreased significantly in both trauma groups after 4 and 6 h, compared to the control group (Figures 3B,D). Lower AH-50 values indicate reduced residual activity of the complement system due to already consumed complement factors after trauma.

### Local Cardiac Levels of C3aR, C5aR1/2 and Local Deposition of C3 Cleavage Products C3b/iC3b/C3c in Multiply Injured Pigs After 6 h

**Figure 4:**
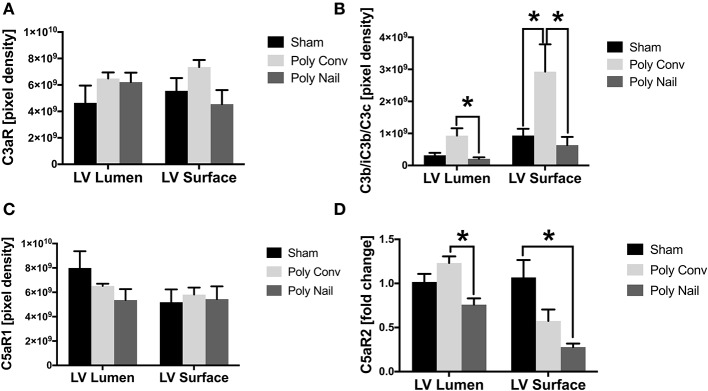
Local cardiac expression of C3a receptor (C3aR), C5a receptors 1 and 2 (C5aR1/C5aR2) and deposition of C3 cleavage products C3b/iC3b/C3c. Pigs received multiple injury, followed by femoral nailing of the fracture (poly nail) or conventional reaming of the fracture (poly conv). Control animals underwent sham-procedure (sham). Tissue of left ventricular lumen and surface were collected 6 h after trauma. Local cardiac expression protein expression of C3aR **(A)**, deposition of C3 cleavage products C3b/iC3b/C3c **(B)** and local expression of C5aR1 **(C)** in control (black), poly conv (light gray), and poly nail (dark gray) pigs, represented as pixel intensity. Local cardiac mRNA expression of *C5aR2* **(D)** in control (black), poly conv (light gray), and poly nail (dark gray) pigs, represented as fold change. Results are presented as mean ± SEM. *n* = 5. *p ≤ 0.05.

Since the multiply injured pigs showed complement activation in serum within 6 h after trauma, we next investigated the local levels of the complement receptors C3aR, C5aR1/2, and the local deposition of the C3 cleavage products C3b/iC3b/C3c in heart tissue. In left ventricles, no significant differences in the expression of the C3aR were observed (Figure 4A). However, the C3aR expression enhanced slightly but not significantly in left ventricular lumen of both polytrauma groups compared to the sham-treated group (Figure 4A). On the ventricular surface, the C3aR expression increased slightly but not significantly in the group with conventional reaming and decreased in the group with femoral nailing, compared to the control group (Figure 4A). The deposition of C3 cleavage products C3b/iC3b/C3c significantly increased in the ventricular lumen of the trauma group with conventional reaming, compared to the group with femoral nailing (Figure 4B). Moreover, the deposition of C3 cleavage products C3b/iC3b/C3c increased significantly on the ventricular surface of the trauma group with conventional reaming, compared to the control group (Figure 4B). In the group with femoral nailing the C3 cleavage products C3b/iC3b/C3c deposition decreased significantly on the ventricular surface compared to the trauma group with conventional reaming (Figure 4B). In the left ventricular lumen, expression of the C5aR1 slightly decreased in both polytrauma groups, compared to the control group (Figure 4C). No differences in C5aR1 expression were observed on the ventricular surface (Figure 4C). The levels of C3aR, C3b/iC3b/C3c, and C5aR1 in porcine samples were determined by IHC due to the restricted availability of tools. The *C5aR2* mRNA expression decreased significantly in the ventricular lumen of the polytrauma group with femoral nailing compared to the group with conventional reaming (Figure 4D). Moreover, the *C5aR2* mRNA expression decreased significantly on the ventricular surface of the polytrauma group with femoral nailing compared to the control group. Also, the *C5aR2* mRNA expression decreased in general in the polytrauma group with conventional reaming on the ventricular surface (Figure 4D). The levels of C5aR2 in porcine samples were determined by qPCR due to the restricted availability of tools.

### Effects of C3a and C5a on Human CMs *in vitro*

**Figure 5:**
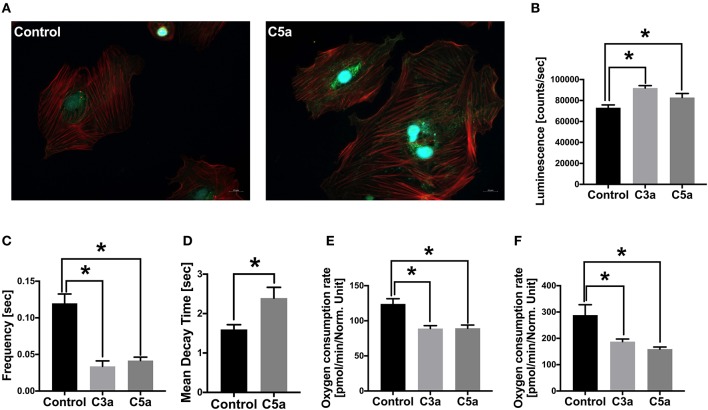
Effects of C3a and C5a on human cardiomyocytes (CMs) *in vitro*. Cellular staining of the C5a receptor 1 (C5aR1) in human CMs in presence of PBS (control) or in presence of C5a **(A)**. Human CMs were treated for 3 h with either PBS or with 10 ng/ml C5a and C5aR1 was stained (green). Cell nuclei were stained with Hoechst (blue) and cytoskeleton was stained with rhodamine-labeled phalloidin (red). Caspase-3/7 activity (counts/s) in human CMs in presence of C3a (light gray) and C5a (dark gray) **(B)**. Frequency of calcium signals (s) of human CMs in presence of C3a and C5a **(C)**. Mean decay time (s) of calcium peaks of human CMs in presence of C5a **(D)**. Basal respiration oxygen consumption rate (OCR in pmol/m in /Norm. unit) of human CMs in presence of C3a and C5a **(E)**. Spare respiratory capacity oxygen consumption rate (OCR in pmol/m in /Norm. unit) of human CMs in presence of C3a and C5a **(F)**. Results are presented as mean ± SEM. *n* = 6. *p ≤ 0.05.

Since the complement system is systemically activated after polytrauma in pigs and acts locally on the heart via its receptors, we next investigated the effects of C3a and C5a on human CMs *in vitro*, trying to investigate the effects of the single complement factors on the cells. First, human CMs were cultured in presence of C5a. After an exposure of 3 h, the C5aR1 of the human CMs was translocated from the surface of the CMs into the cytosol of the cells. In parallel, the intracellular fraction of the C5aR1 increased in presence of C5a in the human CMs (Figure 5A). Furthermore, the Caspase 3/7 activity significantly enhanced in CMs in presence of C5a and C3a, compared to the control group (Figure 5B).

### Calcium Signaling and Metabolic Alterations of Human CMs in Presence of C3a or C5a

Next, we investigated the calcium signaling of the human CMs in presence of C3a or C5a. Calcium is the key signaling element in CMs and is crucial for cardiac function. In CMs, calcium transport includes Ca^2+^ cycling between the cytosol and the extracellular space as well as Ca^2+^ cycling between the cytosol and the intracellular calcium stores, the sarcoplasmic reticulum (SR). During depolarization of the cell membrane, the L-type Ca^2+^ channels in the plasma membrane are activated, allowing Ca^2+^ entering the cytosol, raising local Ca^2+^ concentrations. The local increase of Ca^2+^ triggers activation of further Ca^2+^ channels in the SR membrane. Due to the additional Ca^2+^ release from the SR, the local as well as the global systolic Ca^2+^ levels increase. The increased Ca^2+^ concentrations induce contraction of the CMs via conformational changes in the troponin-tropomyosin complex, allowing the myofilaments actin and myosin to slide past one another (38, 39). For relaxation, the Ca^2+^ has to be removed from the cytosol and is transported back into the SR by the sarcoplasmic-/endoplasmic reticulum ATPase (SERCA) and into the plasma membrane via the sodium-calcium exchanger (NCX). The Ca^2+^ cycling process is induced by extra- or intracellular stimuli, affecting the activity state of Ca^2+^-handling proteins (39). In the present study, the intracellular calcium was labeled with a fluorescent calcium indicator and calcium signaling of the CMs was analyzed by fluorescence microscopy. The frequency of the calcium signals decreased significantly in the CMs after exposed to C3a or C5a (Figure 5C). Moreover, the mean decay time of the single calcium peaks increased significantly in presence of C5a (Figure 5D). Furthermore, the basal mitochondrial respiration decreased significantly in presence of C3a and C5a, compared to the control group (Figure 5E) and the mitochondrial spare respiratory capacity decreased significantly (Figure 5F).

### Cellular Gene Expression in Human CMs in Presence of C3a or C5a

**Figure 6:**
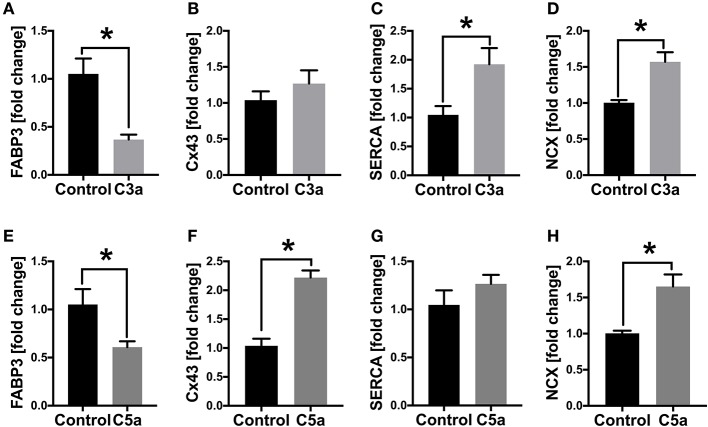
mRNA expression (fold change) of cardiac structure- and gap junction proteins in human cardiomyocytes (CMs) treated with C3a or C5a. Cells were treated for 6 h with either 500 ng/ml C3a or with 10 ng/ml C5a and mRNA expression of *fatty acid binding protein 3 (FABP3)* **(A,E)**, *connexin43* **(B,F)**, *sarcoplasmic/endoplasmic reticulum ATPase (SERCA2a)* **(C,G)** and *sodium-calcium exchanger (NCX)* **(D,H)** were determined. Results are presented as mean ± SEM. *n* = 6. *p ≤ 0.05.

Since the calcium signaling was altered in human CMs in presence of C3a and C5a, we next investigated the gene expression of the gap junction protein Cx43 as well as of the calcium handling proteins *SERCA2a* and *NCX*. In presence of C3a, the *SERCA2a* and *NCX* mRNA expression increased significantly in human CMs, whereas the *Cx43* mRNA expression was not affected (Figures 6B–D). In presence of C5a, the mRNA expression of *Cx43* was significantly increased, as well as the mRNA expression of NCX (Figures 6F,H). The mRNA expression of *SERCA2a* was not affected in presence of C5a (Figure 6G). In presence of C3a and C5a the mRNA expression of *heart fatty acid binding protein (FABP3/HFABP)* decreased significantly in the human CMs (Figures 6A,E).

## Discussion

In the present study, we demonstrated an activation of the complement system in multiply injured pigs within the first 6 h after trauma, which is in accordance with previous studies in humans (3, 40). Thereby, the complement activation resulted in impaired cardiac function of the pigs after trauma. The complement activation was mediated via both, classical and alternative pathway. The complement activation after multiple trauma was probably triggered simultaneously but via different activation cascades. In the present study, the early complement activation might be rapidly induced via the alternative pathway rather than the classical pathway, since the AH-50 dropped significantly directly after trauma as well as after 4 and 6 h in both polytrauma groups, which is in accordance to earlier studies (14). This rapid induction of the alternative pathway might be due to the antibody-independent activation of this pathway. The alternative pathway is immediately activated after binding of danger signals from exogenous pathogens but also from damaged tissue, which is the situation after multiple trauma (41, 42). Furthermore, the CH-50 of the polytrauma group with conventional reaming dropped significantly 6 h after trauma, confirming the complement activation also via the classical pathway. In contrast to the alternative pathway, the activation of the classical pathway is antibody-dependent, which might explain the significant drop of the CH-50 only after 6 h (41). Moreover, the complement activation might correlate with the tissue damage and with the release of bone marrow debris into the circulation reflected by the invasiveness of the fracture provision during both, classical- and alternative pathway. Nevertheless, the activation of the complement system might be also mediated via a third pathway, the lectin pathway. The activation of this pathway is induced when mannose-binding lectin (MBL) binds mannose containing surface proteins on pathogenic surfaces (41). The activation of the complement system via the lectin pathway was not examined, wherefore we cannot exclude complement activation via this pathway in the present study. The polytrauma group, which received conventional reaming showed the highest activation of the complement system. Correlations between enhanced inflammation in general and femoral reaming were also demonstrated previously in humans and were accordingly associated with an increased release of tumor necrosis factor (TNF), interleukin (IL)-6 and IL-10 after fracture treatment (20, 43, 44). The intramedullary nailing in multiply injured patients was described as a less invasive method for fracture treatment (45). Nevertheless, femoral nailing and femoral reaming in multiply injured patients was associated with increased inflammation, occurring as “second hit” after trauma (20, 46). During the “second hit” response, invasive surgical interventions may induce systemic inflammation, leading to systemic inflammatory response syndrome (SIRS), ARDS, ALI, MODS, MOF, and finally to death of the trauma patients (20, 47). Summarized, in the present study we demonstrated that the fracture provision with reamed intramedullary nailing induced an enhanced activation of the complement system after multiple trauma, compared to non-reamed femoral nailing. This increased complement activation might contribute to the “second hit” response after multiple trauma. Accordingly, the choice of fracture treatment might imply the clinical outcome of critically injured patients with respect to the development of SIRS, ARDS, ALI, MODS, and MOF. Consequently, the selection of the treatment strategy might be crucial for survival of the critically injured patients.

The early activation of the complement system may also affect the heart *in vivo*, which was confirmed in the present study by alterations in the left ventricular expression of C3aR, C5aR1/2, and by changes in the deposition of C3 cleavage products C3b/iC3b/C3c, early after trauma. The C3aR expression was enhanced slightly but not significantly in the left ventricles of both polytrauma groups. In humans with non-ischemic heart failure, increased C3aR expression was associated with enhanced cardiac inflammation and progressive heart failure (48). Interestingly, the C3aR expression may correlate with the invasiveness of fracture treatment, indicating augmented inflammation and complement activation during femoral reaming (20). However, in the acute inflammatory phase during ischemia-reperfusion injuries, C3aR activation was shown to reduce the inflammatory response through prevention of leucocyte mobilization (49, 50). In the present study, the deposition of C3 cleavage products C3b/iC3b/C3c were strongly enhanced in the lumen of the left ventricles as well as on the surface of the polytrauma group with conventional reaming, correlating with the invasiveness of the fracture treatment, according to earlier studies (20). Interestingly, in the group with femoral nailing the deposition of C3 cleavage products C3b/iC3b/C3c was significantly decreased on the ventricular lumen and surface, indicating less inflammatory response during trauma (51). An increased amount of deposited C3 cleavage products C3b/iC3b/C3c induces the second inflammatory phase after trauma, resulting in cardiac failure and death (52, 53). In the present study, the expression of C5aR1 was slightly decreased in the lumen of the left ventricle after trauma in both polytrauma groups, which was also described previously after 72 h in an experimental polytrauma model in pigs and after 24 h in an experimental blunt chest trauma model in rats (54, 55). The decreased expression of C5aR1 after trauma was associated with internalization of the receptor, triggered by C5a, which was further associated with CMs dysfunction and compromised cardiac function (25, 56). In contrast, C5aR1 is upregulated during burn injury and after cecal ligature and puncture-induced (CLP) sepsis, mediating complement-induced cardio-depressive effects (25, 28, 57). In the present study, the C5aR1 was slightly upregulated on the surface of the left ventricle of the polytrauma group with femoral reaming, which is in accordance with the above-mentioned studies. Further, C5a elevation during fracture treatment seems to induce a second inflammatory hit after experimental trauma (46). In the present study, the ventricular luminal mRNA expression of the *C5aR2* decreased significantly in the trauma group with femoral nailing, compared to the group with conventional reaming. Interestingly, the mRNA expression of *C5aR2* decreased in both trauma groups on the ventricular surface, compared to sham treated animals. During the inflammatory condition of CLP-sepsis, C5a-C5aR2 interactions were shown to induce excessive amount of cytosolic ROS and [${\text{Ca}}_{\text{i}}^{2+}$] in CMs. Further, the absence of the C5aR2 improves heart function in CLP mice (28, 58). Therefore, the here shown posttraumatic downregulation of the *C5aR2* expression in the heart tissue might even be protective for the heart function during complement activation. The differences in the local cardiac expression of C3aR, C5aR1, C5aR2 and in the deposition of C3 cleavage products C3b/iC3b/C3c might be due to inflammatory effects on the cardiac surface. In the present study, we showed that the complement system is activated in pigs early after polytrauma, acting on the heart via its receptors. Also, the local cardiac complement activation correlated with invasiveness of the fracture treatment. Consequently, the selection of the adequate fracture provision might imply the post-traumatic cardiac function. During early complement activation, the anaphylatoxins C3a and C5a are systemically released. Therefore, we investigated the effects of C3a and C5a on human CMs *in vitro*. In the present study we could show that the C5a was actively internalized by the human CMs via the C5aR1. Thereby, the C5aR1 might be endocytosed during this process, which is in accordance with earlier studies (56, 59). After internalization, the C5aR1 was predominantly localized in tubulo-vesicular structures and around the nucleus, which is in accordance with our observations (60). The increased intracellular fraction of C5aR1 in the human CMs confirmed this assumption as well. C5a was shown to disturb cellular calcium homeostasis and electrophysiological functions and induces defects in CMs contractility and relaxation (28–30).

In the present study, the Caspase-3/7 activity significantly increased in human CMs in presence of C3a and C5a *in vitro*, indicating somehow an enhanced cellular apoptosis. Increased cellular apoptosis in presence of C3a was already demonstrated in previous studies. In these studies, caspase-11 expression was induced by the carboxypeptidase B1 (Cpb1)-C3-C3aR pathway by increasing mitogen-activated protein kinase (MAPK) activity and downstream processing of toll-like receptor 4 (TLR4), resulting in cell death (61). Moreover, C3aR is required for upregulation of caspase-4 and 5 in human macrophages, correlating with cell death and worse outcome of septic patients (61). In contrast, C5a was described to protect against apoptosis by inhibiting MAPK-mediated regulation of caspase-3 cascades in neurons (62). Furthermore, C5a inhibits caspase-9 activity in neutrophils via the phosphatidylinositol-3 kinase (PI-3K)/Akt and via the extracellular signal-regulated kinase (EKR) pathway (63, 64), which is not in agreement with the results in our study. However, the enhanced caspase 3/7 activity might be an effect of the CMs to the exposure of C3a and C5a *in vitro*. We could not detect any apoptosis in cardiac tissue *in vivo* (data not shown), which might indicate for complement-independent apoptotic effects. However, in order to investigate apoptotic effects during complement activation *in vivo*, further studies are necessary.

In the present study, the calcium signaling of the human CMs was disturbed in presence of C3a and C5a, which was demonstrated previously in isolated rat CMs in presence of C5a and was further associated with cardiac dysfunction (28). Here, the frequency of calcium signals decreased significantly, resulting in slowed cell beat and bradycardia. Moreover, the mean decay time of the single calcium peaks was altered in human CMs in presence of C5a, indicating a disturbed calcium handling by enhanced build-up of [Ca^2+^i], as described previously (28). The disturbed calcium signaling in the human CMs might be induced by increased ROS. Enhanced cytosolic ROS was shown in isolated rat and mouse CMs in presence of C5a or when exposed to CLP-sepsis and was associated with myocardial dysfunction and cardiovascular diseases (65, 66). Contractile dysfunction due to enhanced ROS is mostly induced by modification of different calcium regulatory proteins (29, 30). Alterations in mRNA expression of *SERCA2a* and *NCX* in presence of C3a and C5a in our study confirmed this assumption. Moreover, changes in *SERCA2a* and *NCX* mRNA expression were previously shown during CLP-sepsis and were also associated with impaired cardiac function (28). Alterations in these calcium regulatory proteins were mediated via C5a and its receptors C5aR1/C5aR2 (28). Redox imbalance during C5a as well as during C3a treatment might be induced by the NADPH oxidases Nox1 and Nox2 (67). Finally, we could show that the mitochondrial respiration of human CMs was impaired in presence of C3a and C5a. Thereby, the basal mitochondrial respiration as well as the mitochondrial spare respiratory capacity decreased significantly in the cells when treated either with C3a or with C5a. Detrimental effects of C5a on mitochondrial function were already described in pheocromocytoma-derived PC12 cells. Here, C5a inhibited mitochondrial respiration as well as dehydrogenase and cytochrome c oxidase (COX) activities. Moreover, C5a induced mitochondrial stress and damage in these cells (68). Disruption of mitochondrial membrane potential was also demonstrated previously in isolated rat CMs after addition of extracellular histones (27).

Interestingly, in the present study the mRNA expression of the *heart fatty acid binding protein (HFABP/FABP3)* decreased significantly in the human CMs in presence of C3a and C5a. So far, HFABP was primarily used as systemic biomarker for different cardiovascular diseases such as acute myocardial infarction (AMI) (69). Not to forget, HFABP also plays a dominant role in cardiac fatty acid metabolism by promoting the uptake of Long-chain free fatty acids (LCFA) into the cytoplasm of CMs, by delivering the LCFA to the outer membrane of mitochondria and by accelerating the dissociation of LCFA from albumin (70–72). The downregulation of *HFABP* gene expression might indicate for a reduction of the cardiac utilization of LCFA, switching to a condition of enhanced cellular glycolysis, which was also shown previously after experimental multiple trauma (73). This metabolic switch is also called myocardial hibernation and was demonstrated previously in the septic heart (74). Myocardial hibernation is characterized by enhanced expression of the glucose transporter 4 (GLUT4), preventing cardiac cell damage and LV-dysfunction in the injured hypoxic myocardium by enhancing the glucose uptake (75–77).

In summary, we were able to demonstrate an early activation of the complement system in multiply injured pigs within the first 6 h after trauma, which impair the cardiac function *in vivo*. For the first time we could show differential immune response based on surgical treatment strategy (reamed vs. non-reamed intramedullary nailing), which might contribute to the “second hit” response after trauma. Consequently, the selection of the fracture treatment strategy might imply the overall outcome of the multiply injured patients. The anaphylatoxins C3a and C5a acted detrimentally on human CMs *in vitro* by enhancing cellular apoptosis and by disrupting calcium signaling and mitochondrial respiration. Finally, changes in *HFABP* gene expression suggested that CMs could undergo metabolic switch to myocardial hibernation during complement activation resulting from multiple trauma.

## Data Availability Statement

The datasets generated for this study are available on request to the corresponding author.

## Ethics Statement

The animal study was reviewed and approved by Cantonal Veterinary Department, Zurich, Switzerland, under license no. ZH 138/2017.

## Author Contributions

IL, BW, FG, RP, PC, SH, ML, NC, HS, MH-L, H-CP, and MK designed the research. IL, BW, MB, GF, SH, ML, and NC performed the research. IL wrote the paper. IL and MK analyzed the data. FG, RP, PC, HS, H-CP, and MK contributed new reagents or analytic tools. All authors made substantial contributions to conception and design of the study, participated in drafting the article and gave final approval of the version to be published.

### Conflict of Interest

The authors declare that the research was conducted in the absence of any commercial or financial relationships that could be construed as a potential conflict of interest.
